# Improving surgical antibiotic prophylaxis in obstetric and gynaecological surgery: a prospective two-cycle quality-improvement clinical audit in a North African tertiary hospital^☆^

**DOI:** 10.1016/j.infpip.2026.100600

**Published:** 2026-09-23

**Authors:** Ikram Hajjar, Fatma Sellami, Mariem Znati, Aymen Abbassi, Hallouma Ben Fredj, Alia Jebri, Mariem Rahoui, Cyrine Lahmar, Mohamed Abidine, Mariem Garci, Ons Mrad, Nabil Mathlouthi

**Affiliations:** a Pharmacy Department, Charles Nicolle Hospital, Tunis, Tunisia; b Department of Anaesthesiology and Intensive Care, Charles Nicolle Hospital, Tunis, Tunisia; c Department of Gynaecology and Obstetrics, Charles Nicolle Hospital, Tunis, Tunisia

**Keywords:** Surgical antibiotic prophylaxis, Antimicrobial stewardship, Infection prevention, Quality improvement, Surgical site infection, Gynaecological surgery

## Abstract

**Background:**

Inappropriate surgical antibiotic prophylaxis (SAP), particularly continuation beyond the recommended prophylactic period, increases avoidable antimicrobial exposure and is a key target for infection prevention and antimicrobial stewardship.

**Methods:**

A prospective two-cycle targeted clinical audit evaluated 60 eligible procedures included consecutively during active audit observation periods (30 baseline and 30 postintervention) in an obstetric and gynaecological operating theatre of a North African tertiary hospital. Criteria were based on French Haute Autorité de Santé audit tools and Société Française d’Anesthésie et de Réanimation/ Société de Pathologie Infectieuse de Langue Française recommendations. The primary outcome was SAP duration compliant with procedure-specific guidance. The multi-faceted intervention comprised protocol revision and validation, point-of-care access, multi-disciplinary training, visual reminders, role clarification, and surveillance tools.

**Results:**

Protocol-governance compliance rose from 30% to 100%; allergy documentation from 67% to 100%; documentation of administration modalities from 3% to 37%; and appropriate antibiotic choice from 57% to 90%. Appropriate SAP duration improved from 10% to 53% (*P* < 0.001), while SAP continuation beyond 48 h decreased from 83% to 47% (*P* = 0.006). Documented administration within 60 min before incision decreased from 70% to 47%; this documentation-sensitive indicator was interpreted cautiously because non-compliance could reflect either missing timing information or administration outside the recommended window.

**Conclusion:**

A pragmatic multi-disciplinary intervention improved several SAP process indicators and reduced unnecessary continuation beyond the recommended prophylactic period. Further audit cycles should prioritise reliable timing documentation, sustained reduction of prolonged SAP, and feedback of local surveillance data.

## Highlights

• A two-cycle audit improved surgical antibiotic prophylaxis (SAP) governance.
• Appropriate antibiotic selection increased from 57% to 90%.
• Compliance with recommended prophylaxis duration increased from 10% to 53%.
• Prophylaxis continuing beyond 48 h decreased from 83% to 47%.
• Timing documentation and surveillance feedback require further improvement.

## Introduction

Surgical site infections (SSIs) are among the most frequent healthcare-associated complications after surgery and are associated with prolonged hospitalisation, additional antimicrobial exposure, increased costs, and avoidable morbidity [[1], [2], [3]]. In obstetric and gynaecological surgery, infection risk is influenced by exposure to polymicrobial genital-tract flora, particularly during caesarean delivery, hysterectomy, and procedures involving entry into the uterus or vagina. Patient-related factors, including obesity, diabetes, anaemia, and emergency surgery, may further increase this risk [4].

Surgical antibiotic prophylaxis (SAP) is a key evidence-based intervention for SSI prevention. Its purpose is to ensure adequate antimicrobial concentrations in tissues at the time of incision and throughout the period of potential bacterial contamination. Effective SAP depends on adherence to several critical components: appropriate indication, antibiotic selection, dose, timing before incision, intra-operative redosing when indicated, and postoperative discontinuation, according to procedure-specific recommendations [1,2,5].

Inappropriate SAP, especially continuation beyond the recommended prophylactic period, is also an important antimicrobial-stewardship concern. Prolonged SAP increases antimicrobial exposure without clear additional benefit for most clean or clean-contaminated procedures and may contribute to adverse drug events, selection pressure, and AMR [1,5,6]. This issue is particularly relevant in resource-limited settings where surveillance systems, electronic prescribing, and real-time stewardship support may be limited.

Targeted clinical audits are practical quality-improvement tools because they identify gaps between recommended and actual practice, guide corrective actions, and enable reassessment using predefined criteria. The aim of this study was to evaluate and improve SAP practices in the obstetric and gynaecological operating theatre of a North African tertiary-care university hospital through a prospective two-cycle targeted clinical audit.

## Methods

### Study design and setting

We conducted a prospective two-cycle targeted clinical audit in the obstetric and gynaecological operating theatre of a 900-bed tertiary-care university hospital in Tunis, Tunisia. The project followed a Plan–Do–Check–Act quality-improvement approach and was reported in accordance with the SQUIRE 2.0 (Standards for Quality Improvement Reporting Excellence) guidelines [7,8].

The baseline audit round was conducted from 18^th^ March to 30^th^ March 2024. Corrective measures were implemented between April and December 2024, and the postintervention reassessment was conducted from 24^th^ December to 28^th^ December 2024. Thirty eligible procedures were included consecutively during active observation periods in each round.

The audit sample was predefined as 30 eligible procedures per cycle, included consecutively during active observation periods, in accordance with the Haute Autorité de Santé (HAS; French National Authority for Health) targeted clinical-audit approach [7]. The audit was designed to identify deviations from predefined standards and reassess corrective actions rather than to estimate compliance across the department’s entire annual surgical activity. Annual and audit-period surgical-volume denominators were not prospectively collected as part of the audit dataset and could not be retrospectively reconstructed with sufficient reliability. The difference in calendar duration between the two rounds was partly related to the organisation of data collection. During the baseline round, observation was interrupted on some days because the designated auditor was unavailable. The same trained auditor was deliberately retained for both rounds to minimise interobserver variability in applying the audit criteria. During reassessment, eligible procedures were more concentrated on the auditor’s observation days, allowing the same predefined sample of 30 procedures to be reached over a shorter calendar period. The sampling strategy and target sample size remained unchanged between rounds.

### Audit standards, definitions, and outcome measures

Audit criteria were based on the French HAS targeted clinical-audit methodology and on Société Française d’Anesthésie et de Réanimation (SFAR; French Society of Anaesthesia and Intensive Care Medicine)/Société de Pathologie Infectieuse de Langue Française (SPILF; French-speaking Society of Infectious Diseases) recommendations for antibiotic prophylaxis in surgery and interventional medicine [5,7]. The same audit grids, operational definitions, denominators, and data sources were used in both audit rounds. Data sources included anaesthesia records, surgical records, prescriptions, and audit forms.

The primary process outcome was the proportion of procedures with total SAP duration compliant with the procedure-specific protocol aligned with SFAR/SPILF recommendations. Duration was considered compliant when SAP was stopped at the end of surgery or within 24 h postoperatively, unless a longer duration was explicitly recommended for a specific procedure or justified by the clinical context.

Secondary outcomes included appropriate antibiotic selection, initial-dose compliance, documented administration within 60 min before incision, intra-operative redosing when indicated and evaluable, SAP discontinuation or total duration compliant with the procedure-specific protocol, SAP continuation beyond 48 h, allergy documentation, documentation of administration modalities, and organisational compliance with protocol-governance and surveillance criteria.

Timing compliance was assessed from perioperative documentation, primarily anaesthesia records and operating-room records. This criterion was distinct from the prescription criterion ‘administration modalities specified’, which assessed whether the written prescription included sufficient information on route, dose, administration conditions, and planned duration. Administration within 60 min before incision was counted as documented compliance only when both antibiotic-administration time and incision time were available and supported the criterion. The audit dataset retained a binary compliance result and did not preserve a separate numerical breakdown of administration outside the 60-min window vs non-verifiable timing among non-compliant cases; these situations could therefore not be reliably distinguished retrospectively.

The organisational audit assessed two domains: SAPpro (protocol-governance audit grid), which included 10 criteria related to the existence, validation, updating, accessibility, and content of the SAP protocol; and SAPsur (surveillance audit grid), which included six criteria related to SSI surveillance and antibiotic-consumption monitoring. The practice audit assessed prescription criteria and administration criteria, including written prescription, patient identification, antibiotic name, allergy documentation, administration modalities, prescriber identity, antibiotic choice, timing, dose, redosing, and total SAP duration.

Although the local SAP protocol was revised between baseline and reassessment as part of the intervention, both rounds were assessed using a standardised procedure-specific reference aligned with the same national recommendations. Changes in compliance were therefore interpreted as changes in implementation and practice rather than as changes in the measurement method.

Providing both protocol versions as supplementary material also clarifies that the reassessment evaluated implementation within a quality-improvement cycle, including changes in protocol format, accessibility, multi-disciplinary validation, governance, role clarification, and staff training.

### Participants and eligibility criteria

Eligible cases were clean or clean-contaminated planned surgical procedures according to the Altemeier classification, with an indication for first-intention SAP and no documented pre-existing SSI. Contaminated or dirty procedures, procedures performed in the presence of established infection, incomplete records preventing assessment of the main criteria, and repeated procedures in the same patient were excluded.

Procedure type was summarised for each audit round to assess broad comparability of the case mix (Table I).

**Table I:** Procedure type in the two audit rounds

Characteristic	Baseline round, T1	Post-intervention round, T2	*P-value*
Caesarean delivery	19 (63.3)	16 (53.3)	0.556
Hysterectomy	9 (30.0)	13 (43.3)	
Other gynaecological procedures	2 (6.7)	1 (3.3)	

T1, baseline audit round; T2, postintervention audit round.

Values are *N* (%). The overall *P* value was calculated using the two-sided Fisher–Freeman–Halton exact test.

### Intervention

After analysis of baseline findings, a multi-faceted intervention was implemented between April and December 2024. A gynaecology–obstetrics–specific SAP protocol was revised, dated, version-controlled, and formally validated by gynaecological surgeons, anaesthesiologists, pharmacists, and the hospital antibiotic-stewardship committee. The protocol was then made available in operating rooms, staff areas, and physicians’ offices.

The baseline hospital-wide SAP protocol provisions applicable to obstetric and gynaecological surgery and the full revised multi-disciplinary validated obstetric–gynaecological SAP protocol are provided as Supplementary Files 1 and 2, respectively, in English translation. These documents permit direct comparison of the pre-existing clinical recommendations with the revised protocol and clarify the respective contributions of clinical-content changes, format, accessibility, multi-disciplinary validation, governance, role clarification, training, and surveillance.

Responsibilities were clarified: prescription was assigned to surgeons, whereas administration was assigned to anaesthesiologists. A multi-disciplinary training session was conducted on 16^th^ September 2024 for surgeons, anaesthesiologists, residents, operating-room nurses, and ward nurses. Visual reminders summarising recommended antibiotics, doses, dilution, administration modalities, timing, and special circumstances were displayed in relevant clinical areas.

Specific emphasis was placed on systematic allergy documentation, documentation of route and duration of administration, and recording of both antibiotic-administration time and incision time. An SSI and antibiotic-use surveillance form was introduced in collaboration with the microbiology and stewardship teams. The intervention was directly mapped to baseline gaps identified during the first audit round, with corresponding audit indicators used to assess change between rounds (Table II).

**Table II:** Corrective actions and corresponding audit outcomes

Audit indicator	Corrective action	Baseline, T1 *n/N* (%)	Postintervention, T2 *n/N* (%)	*P* value^a^
SAPpro protocol-governance compliance	Protocol revision, version control, multi-disciplinary validation, and point-of-care access	3/10 (30.0)	10/10 (100.0)	—
SAPsur surveillance compliance	SSI and antibiotic-use surveillance forms and periodic audit planning	2/6 (33.3)	4/6 (66.7)	—
Antibiotic choice compliant with protocol	Protocol display, staff training, and visual reminders	17/30 (56.7)	27/30 (90.0)	0.007
SAP administered when indicated	Prescription assigned to surgeons and administration to anaesthesiologists	29/30 (96.7)	29/30 (96.7)	1.000
Documented administration ≤60 min before incision	Training, visual reminders, and emphasis on recording administration and incision times	21/30 (70.0)	14/30 (46.7)	0.115
Initial dose compliant with protocol	Procedure-specific and patient-specific dose reminders	21/30 (70.0)	27/30 (90.0)	0.104
Total SAP duration compliant with procedure-specific protocol	Education on timely discontinuation and protocol display	3/30 (10.0)	16/30 (53.3)	< 0.001
SAP continuation beyond 48 h	Education on timely discontinuation and protocol display	25/30 (83.3)	14/30 (46.7)	0.006

T1, baseline audit round; T2, postintervention audit round; SAPpro, protocol-governance audit grid; SAPsur, surveillance audit grid; SSI, surgical site infection; SAP, surgical antibiotic prophylaxis.

Values are number compliant/number assessed (%), unless otherwise indicated.

a Procedure-level *P* values were calculated using two-sided Fisher exact tests; SAPpro and SAPsur changes were reported descriptively. The timing variable did not distinguish documented late administration from non-verifiable timing among non-compliant cases.

### Data collection and statistical analysis

Data were collected prospectively from anaesthesia records, surgical records, prescriptions, and audit grids. Compliance was expressed as numbers and percentages. Procedure-level comparisons between the two independent audit samples used two-sided Fisher exact tests because of the small sample size and sparse cells. The overall distribution of procedure types was compared using the two-sided Fisher–Freeman–Halton exact test. Organisational-domain changes in SAPpro and SAPsur were reported descriptively because they represented paired assessments of a fixed set of criteria rather than independent patient-level observations. A two-sided *P* value < 0.05 was considered statistically significant. Statistical calculations were performed using Python version 3.13 and SciPy version 1.17.0 (Python Software Foundation, Wilmington, Delaware, USA).

Because this was a quality-improvement audit based on process indicators, the study was not designed or powered to assess SSI incidence. Any SSI information was therefore interpreted descriptively and not as a comparative clinical outcome.

## Results

### Patient and procedure characteristics

Sixty eligible procedures were included consecutively during active observation periods: 30 in the baseline round and 30 in the postintervention round. Procedure type was broadly similar between rounds (Table I).

### Baseline organisational audit

At baseline, the organisational audit identified important gaps in the SAP implementation framework. Although a written SAP protocol was available in the department, only three of 10 SAPpro criteria were compliant, corresponding to 30% global protocol compliance. The protocol was not dated, was not formally co-signed by surgeons and anaesthesiologists, and did not include a defined revision schedule. Responsibilities for prescription and administration were also not clearly assigned.

The surveillance component showed limited compliance. Only two of six SAPsur criteria were met, corresponding to 33% compliance. No structured SSI surveillance system was in place, and antibiotic consumption was not systematically monitored or reported to frontline teams.

### Baseline prescription audit

Baseline prescription performance was satisfactory for several administrative criteria. Written prescription, prescription date, complete patient identification, antibiotic name, and prescriber identity were documented in 29 of 30 cases (97%). Allergy status was documented in 20 cases (67%). Documentation of administration modalities was uncommon, with compliance in only one of 30 cases (3%).

Thus, the main prescription gap concerned insufficient specification of administration modalities in the written prescription, including route, dose, administration conditions, or planned duration. This prescription criterion was distinct from the timing indicator, which was assessed separately using perioperative anaesthesia and operating-room records. Compliance with individual prescription criteria at baseline and after intervention is shown in Table III.

**Table III:** Compliance with surgical antibiotic prophylaxis prescription criteria at baseline and after intervention

Prescription criterion	Baseline, T1 *n/N* (%)	Postintervention, T2 *n/N* (%)	*P* value^a^
Written prescription	29/30 (96.7)	29/30 (96.7)	1.000
Dated prescription	29/30 (96.7)	29/30 (96.7)	1.000
Complete patient identification	29/30 (96.7)	29/30 (96.7)	1.000
Antibiotic name specified	29/30 (96.7)	29/30 (96.7)	1.000
Allergy checked and recorded	20/30 (66.7)	30/30 (100.0)	< 0.001
Administration modalities specified in written prescription	1/30 (3.3)	11/30 (36.7)	0.002
Prescriber identity recorded	29/30 (96.7)	29/30 (96.7)	1.000

SAP, surgical antibiotic prophylaxis; T1, baseline audit round; T2, postintervention audit round.

Values are number compliant/number assessed (%).

a Two-sided Fisher exact test.

### Baseline administration audit

At baseline, SAP was administered when indicated in 29 of 30 cases (97%). Antibiotic choice was compliant with the protocol in 17 of 30 cases (57%). Documented administration within 60 min before incision was recorded in 21 of 30 cases (70%) using perioperative anaesthesia and operating-room records. The binary audit variable did not retain a separate breakdown of the remaining cases into documented late administration and non-verifiable timing. Initial-dose compliance was observed in 21 of 30 cases (70%).

The major administration gap concerned inappropriate continuation of SAP after surgery. Total SAP duration was compliant with the procedure-specific recommendations in three of 30 cases (10%). SAP continued beyond 48 h in 25 of 30 cases (83%), indicating frequent extension beyond the recommended prophylactic period.

### Postintervention organisational audit

After implementation of corrective measures, organisational compliance improved substantially. SAPpro compliance increased descriptively from three of 10 criteria at baseline to 10 of 10 criteria at reassessment (30%–100%). The updated protocol was dated, validated, accessible, adapted to current recommendations, and supported by clearly defined responsibilities.

SAPsur compliance improved from two of six to four of six criteria, corresponding to an increase from 33% to 67%. Newly implemented elements included periodic SAP audit planning and structured monitoring of SSI and antibiotic-use indicators. However, systematic feedback of surveillance data to frontline teams and structured retrospective analysis of SSI cases remained incomplete.

### Postintervention prescription audit

Prescription compliance remained high for written prescription, prescription date, patient identification, antibiotic name, and prescriber identity, with 29 of 30 cases compliant in both rounds. Allergy documentation improved from 20 of 30 cases (67%) to 30 of 30 cases (100%; *P* < 0.001). Documentation of administration modalities improved from one of 30 cases (3%) to 11 of 30 cases (37%; *P* = 0.002), but remained suboptimal. Detailed prescription criteria are presented in Table III.

### Postintervention administration audit

Several administration criteria improved after the intervention. Administration when indicated remained stable at 29 of 30 cases (97%). Antibiotic choice according to protocol improved from 17 of 30 cases (57%) to 27 of 30 cases (90%; *P* = 0.007). Most prescriptions in the second audit round used cefazolin as the first-line agent, with alternatives reserved for documented contraindications or allergy, in accordance with current recommendations [5].

Documented administration within 60 min before incision decreased from 21 of 30 cases (70%) to 14 of 30 cases (47%; *P* = 0.115). Because the binary audit variable did not distinguish documented administration outside the recommended window from non-verifiable timing among non-compliant cases, the apparent decrease cannot be attributed solely to poorer documentation or solely to deterioration in clinical timing. It should therefore be interpreted as an unresolved documentation-sensitive finding.

Initial-dose compliance increased from 21 of 30 cases (70%) to 27 of 30 cases (90%; *P* = 0.104). Intra-operative redosing was not analysed as a comparative outcome because no procedure in the second audit round met the prespecified indication for redosing and the available dataset did not provide a denominator restricted to procedures requiring redosing in both rounds.

Compliance with total SAP duration, the primary process outcome, improved from three of 30 cases (10%) to 16 of 30 cases (53%; *P* < 0.001). Procedures with SAP continuing beyond 48 h decreased from 25 of 30 cases (83%) to 14 of 30 cases (47%; *P* = 0.006). Administration criteria and their corresponding corrective actions are presented in Table II.

### Descriptive SSI and antibiotic-use surveillance

No formal comparison of SSI incidence was performed because the audit was not designed or powered for clinical outcomes. The intervention introduced SSI and antibiotic-use surveillance forms to support future feedback; however, SSI data were not sufficiently complete or standardised to support comparative or safety conclusions.

## Discussion

This prospective two-cycle targeted clinical audit identified important gaps in the implementation of SAP in obstetric and gynaecological surgery and showed that a pragmatic multi-disciplinary intervention improved several process indicators. Beyond the observed improvement in compliance, the main message is that SAP quality depends not only on the existence of written recommendations, but also on governance, accessibility, multi-disciplinary ownership, clear allocation of responsibilities, and reliable documentation. This is consistent with the principles of targeted clinical audit and quality-improvement reporting, which emphasise measurement of practice gaps, implementation of corrective actions, and reassessment using predefined criteria [7,8].

One important finding was the gap between the presence of a written protocol and its effective implementation. At baseline, the protocol was available but was not sufficiently embedded in routine practice. The intervention addressed this implementation gap through formal validation, version control, point-of-care access, staff training, visual reminders, and clarification of responsibilities between surgical and anaesthesia teams. This suggests that improving SAP requires an implementation strategy rather than passive dissemination of guidelines alone. Multi-disciplinary validation was particularly important because SAP involves several professional groups and depends on co-ordinated decisions regarding indication, prescription, timing, administration, and discontinuation [5].

The reduction in SAP continuation beyond the recommended prophylactic period is particularly relevant from an antimicrobial-stewardship perspective. Current recommendations emphasise that SAP should provide adequate antimicrobial concentrations during the period of bacterial contamination, while avoiding unnecessary exposure after surgery [1,5]. Continued antibiotic administration beyond the recommended prophylactic period has not been shown to provide additional preventive benefit for most clean or clean-contaminated procedures and may contribute to adverse drug events, selection pressure, and AMR [1,2,5,6]. The persistence of prolonged SAP in a substantial proportion of cases after the intervention indicates that prescribing habits, perceived infection risk, and limited feedback using local outcome data may remain important barriers.

The apparent decrease in documented timing compliance should be interpreted cautiously. The timing indicator was derived from perioperative anaesthesia and operating-room records and was separate from the prescription criterion ‘administration modalities specified’. The latter assessed whether the written order included practical administration details, whereas the timing indicator required documented antibiotic-administration and incision times. Because the audit dataset retained only a binary compliance result, documented late administration could not be reliably separated from non-verifiable timing among non-compliant cases. Measured timing compliance may therefore reflect both documentation quality and actual clinical practice, highlighting the need for mandatory time-stamped fields in future cycles.

The intervention was deliberately low-cost and feasible in a resource-limited environment. Its main components – protocol standardisation, multi-disciplinary validation, education, visual reminders, role clarification, and introduction of surveillance tools – do not require advanced electronic systems. This makes the approach potentially transferable to other surgical specialties and comparable hospitals, provided that local teams adapt the protocol to their own procedure mix, AMR context, and organisational constraints. However, such transferability should be assessed through repeated local audit cycles rather than assumed from a single before-and-after evaluation [7,8].

This study has several methodological limitations that should be considered when interpreting the findings. First, it was conducted in a single obstetric and gynaecological operating theatre of one tertiary-care hospital, which may limit generalisability to other surgical specialties, hospitals, or healthcare systems. Second, only 30 procedures were included in each audit round. This sample size is consistent with the targeted clinical-audit methodology recommended by the HAS, but it limits statistical precision and increases the possibility that some differences may have been influenced by random variation or small changes in case mix [7].

Third, the two audit periods differed in calendar duration and timing within the year. The baseline round required 13 days to include 30 eligible procedures, whereas the reassessment round required 5 days. During the baseline round, observation was interrupted on some days because the designated auditor was unavailable. Retaining the same trained auditor across both rounds reduced interobserver variability, but intermittent availability extended the baseline calendar window. Eligible procedures were also more concentrated on the auditor’s observation days during reassessment. Because annual and audit-period surgical-volume denominators were not collected, the contribution of differences in procedure density could not be quantified. These organisational differences may have influenced temporal sampling; therefore, the before-and-after comparison should be interpreted as a quality-improvement reassessment rather than as a controlled effectiveness estimate, in line with the scope of targeted audit and SQUIRE-oriented quality-improvement reporting [7,8].

Fourth, the local SAP protocol was revised, formatted, validated, and disseminated as part of the intervention. Although both audit rounds were assessed using the same procedure-specific reference aligned with SFAR/SPILF recommendations, the intervention combined several components: protocol revision, improved accessibility, multi-disciplinary validation, training, visual reminders, role clarification, and surveillance tools [5]. Consequently, the audit cannot determine which individual component had the greatest effect, nor can it fully separate the effect of clinical-content clarification from the effect of implementation and governance.

Fifth, a Hawthorne effect is possible because staff awareness of audit activities may have influenced prescribing, administration, or documentation behaviour during observation periods. This effect may have led to overestimation of compliance during reassessment, particularly for documentation-sensitive indicators. The available audit dataset also did not support a valid between-round comparison of intra-operative redosing because it lacked a consistent denominator restricted to procedures in which redosing was indicated. Finally, the audit focused on process indicators and was not designed or powered to assess SSI incidence or other patient-level outcomes. SSI data were not sufficiently complete or standardised for comparative analysis; the findings should therefore not be interpreted as evidence of clinical effectiveness in reducing SSI, which would require larger studies with standardised postoperative surveillance [[1], [2], [3]].

Despite these limitations, this audit suggests that a structured multi-disciplinary approach can improve important SAP processes in obstetric and gynaecological surgery. Future audit cycles should prioritise reliable recording of antibiotic administration and incision times, further reduction of SAP continuation beyond the recommended prophylactic period, and routine feedback of SSI and antibiotic-use indicators to frontline teams.

This prospective two-cycle targeted clinical audit identified important opportunities to improve SAP in obstetric and gynaecological surgery. A multi-faceted intervention combining protocol revision, multi-disciplinary validation, improved accessibility, staff training, role clarification, and surveillance tools improved organisational compliance, antibiotic choice, allergy documentation, and adherence to recommended SAP duration, while reducing continuation beyond 48 h. Persistent gaps in documentation and duration compliance support the need for repeated audit cycles and regular feedback. This approach provides a feasible model for strengthening perioperative infection prevention and antimicrobial stewardship in a North African tertiary-care hospital.

## CRediT authorship contribution statement

**Ikram Hajjar:** Methodology, Investigation, Data curation. **Fatma Sellami:** Validation, Project administration, Methodology, Investigation, Conceptualization. **Mariem Znati:** Data curation. **Aymen Abbassi:** Supervision. **Hallouma Ben Fredj:** Writing – review & editing, Writing – original draft, Supervision. **Alia Jebri:** Validation. **Mariem Rahoui:** Supervision. **Cyrine Lahmar:** Data curation, Conceptualization. **Mohamed Abidine:** Data curation, Conceptualization. **Mariem Garci:** Visualization, Supervision. **Ons Mrad:** Resources, Project administration, Methodology. **Nabil Mathlouthi:** Visualization, Validation, Supervision, Software.

## Data availability

The deidentified data underlying this article are available from the corresponding author upon reasonable request and in accordance with institutional and ethical regulations.

## Ethical approval

This project was a service-evaluation/quality-improvement audit of routine practice based solely on anonymised data and without modification of patient care. Under the institutional policy of the Infection Control Committee of Charles Nicolle Hospital, it was deemed exempt from formal research-ethics review and individual informed consent. The project was conducted in accordance with applicable institutional requirements and the principles of the Declaration of Helsinki.

## Funding sources

This research did not receive any specific grant from funding agencies in the public, commercial, or not-for-profit sectors.

## Conflict of interest statement

The authors declare that they have no known competing financial interests or personal relationships that could have appeared to influence the work reported in this paper.

## Appendix A. Supplementary data

Supplementary File 1 Baseline hospital-wide surgical antibiotic prophylaxis protocol: general rules and obstetric–gynaecological provisions, English translation.

Supplementary File 2 Revised multi-disciplinary validated obstetric and gynaecological surgical antibiotic prophylaxis protocol, Version 01, English translation.
