# Supplementary material for: Improving surgical antibiotic prophylaxis in obstetric and gynaecological surgery: a prospective two-cycle quality-improvement clinical audit in a North African tertiary hospital
Source: Infect Prev Pract. 2026 Sep 23;8(4):100600. doi: 10.1016/j.infpip.2026.100600 (PMC13693070; doi:10.1016/j.infpip.2026.100600)
Supplement: Supplementary file 1 [file mmc1.docx]

**SUPPLEMENTARY FILE 1**

**Baseline Written Surgical Antibiotic Prophylaxis Protocol**

*English translation of the hospital-wide protocol provisions applicable to obstetric and gynaecological surgery*

| Institution | Charles Nicolle Hospital, Tunis, Tunisia |
| --- | --- |
| Clinical area | Obstetrics and gynaecology |
| Purpose | English-language supplementary material accompanying the quality-improvement report |
| Translation note | The clinical content, doses, routes, timing, and redosing instructions were translated from the original French institutional document. Formatting was standardised for readability; the original signed French document remains the source record. |

# 1. Source and scope

At baseline, the department used a hospital-wide surgical antibiotic prophylaxis protocol adapted from the 2018 recommendations of the French Society of Anaesthesia and Intensive Care Medicine. The present supplementary file reproduces the general rules and the obstetric-gynaecological section that were applicable to the audited operating theatre.

# 2. General prescribing rules

- Use prophylaxis for selected clean procedures and for clean-contaminated procedures according to the Altemeier classification.
- Identify the expected bacterial target according to the type of surgery, the patient’s endogenous flora, and the local microbiological ecology.
- Use an initial dose approximately twice the usual therapeutic unit dose; beta-lactam doses are increased in patients with obesity according to the bariatric-surgery recommendations.
- Adapt prophylaxis in patients colonised with multidrug-resistant organisms and in patients undergoing early reoperation for a non-infectious indication.
- Radiotherapy, chemotherapy, corticosteroid therapy, poorly controlled diabetes, advanced age, obesity, or low body weight do not by themselves justify changing the usual target-organism regimen.
- Use a single dose whenever possible; prophylaxis may continue for up to 24 hours and only exceptionally for 48 hours.
- Administer prophylaxis approximately 30 minutes before incision, including before planned caesarean delivery.
- When vancomycin is indicated, complete the infusion by the start of surgery and preferably 30 minutes before incision.
- For prolonged oncological surgery requiring repeated amoxicillin-clavulanate, alternate amoxicillin with amoxicillin-clavulanate to limit daily clavulanate exposure and adjust dosing to renal function.

# 3. Target organisms

Staphylococcus aureus and enteric flora when a skin incision is performed; vaginal flora, including aerobic and anaerobic polymicrobial flora, when the uterus or vagina is entered.

# 4. Procedure-specific recommendations

| **Procedure / indication** | **Recommended agent** | **Initial dose / route** | **Redosing and duration** |
| --- | --- | --- | --- |
| Hysterectomy (abdominal or vaginal route); laparoscopic gynaecological surgery | Cefazolin or cefuroxime Beta-lactam allergy: clindamycin + gentamicin | Cefazolin 2 g IV slowly Cefuroxime 1.5 g IV slowly Clindamycin 900 mg IV slowly + gentamicin 5 mg/kg IV | Cefazolin: repeat 1 g if surgery >4 h Cefuroxime: repeat 0.75 g if surgery >2 h Clindamycin/gentamicin: single dose |
| Caesarean delivery | Cefazolin or cefuroxime Beta-lactam allergy: clindamycin | Cefazolin 2 g IV slowly Cefuroxime 1.5 g IV slowly Clindamycin 900 mg IV slowly | Single dose |
| Mastectomy; breast reconstruction and/or breast plastic surgery | Cefazolin or cefuroxime Beta-lactam allergy: clindamycin ± gentamicin | Cefazolin 2 g IV slowly Cefuroxime 1.5 g IV slowly Clindamycin 900 mg IV slowly; gentamicin 5 mg/kg IV when indicated | Cefazolin: repeat 1 g if >4 h Cefuroxime: repeat 0.75 g if >2 h Clindamycin: repeat 600 mg if >4 h; gentamicin single dose |
| Prolapse surgery, any approach, only when prosthetic material is implanted (promontofixation, implant, or sling) | Amoxicillin-clavulanate Beta-lactam allergy: metronidazole + gentamicin | Amoxicillin-clavulanate 2 g IV Metronidazole 1 g IV + gentamicin 5 mg/kg IV | Amoxicillin-clavulanate: repeat 1 g if >2 h Alternative regimen: single dose |
| Manual placental removal without postpartum haemorrhage | Cefazolin Beta-lactam allergy: clindamycin | Cefazolin 2 g IV slowly Clindamycin 900 mg IV slowly | Single dose |
| Uterine revision without postpartum haemorrhage, with risk factors such as gestational age <37 weeks, fever, or prolonged rupture of membranes >18 h; postpartum haemorrhage | Cefazolin Beta-lactam allergy: clindamycin | Cefazolin 2 g IV slowly Clindamycin 900 mg IV slowly | Single dose |
| Uterine revision without postpartum haemorrhage and without the above risk factors | No prophylaxis | — | — |
| Diagnostic or exploratory laparoscopy without vaginal or digestive incision; hysteroscopy; hysterosalpingography; endometrial biopsy; in-vitro fertilisation; insertion of an intrauterine device; termination of pregnancy; simple breast lumpectomy | No prophylaxis | — | — |

# 5. Baseline protocol characteristics relevant to the audit

- The protocol was hospital-wide rather than dedicated exclusively to obstetric and gynaecological surgery.
- It contained procedure-specific clinical recommendations but did not provide the later version-control, multidisciplinary validation, role-allocation, dissemination, and surveillance framework introduced during the quality-improvement intervention.
- The baseline recommendations were used as the written departmental reference available before implementation of the revised protocol.
