# Supplementary material for: Improving surgical antibiotic prophylaxis in obstetric and gynaecological surgery: a prospective two-cycle quality-improvement clinical audit in a North African tertiary hospital
Source: Infect Prev Pract. 2026 Sep 23;8(4):100600. doi: 10.1016/j.infpip.2026.100600 (PMC13693070; doi:10.1016/j.infpip.2026.100600)
Supplement: Supplementary file 2 [file mmc2.docx]

**SUPPLEMENTARY FILE 2**

**Revised Multidisciplinary Validated Surgical Antibiotic Prophylaxis Protocol**

*English translation of the obstetric and gynaecological protocol, Version 01*

| Institution | Charles Nicolle Hospital, Tunis, Tunisia |
| --- | --- |
| Clinical area | Obstetrics and gynaecology |
| Purpose | English-language supplementary material accompanying the quality-improvement report |
| Translation note | The clinical content, doses, routes, timing, and redosing instructions were translated from the original French institutional document. Formatting was standardised for readability; the original signed French document remains the source record. |

# Document control

| Original title | Protocole antibioprophylaxie en gynécologie et en obstétrique |
| --- | --- |
| Version | 01 |
| Drafted by | Cyrine Lahmar, resident in anaesthesiology and intensive care; Ons Mrad, resident in obstetrics and gynaecology |
| Draft date | 15 July 2024 |
| Validated by | Dr Alia Jebri, Head of Anaesthesiology and Intensive Care; Dr Nabil Mathlouthi, Head of Gynaecology A - validation dated 8 August 2024 |

# 1. Purpose

To establish a surgical antibiotic prophylaxis protocol for gynaecological and obstetric surgery in order to reduce the incidence of surgical-site infection.

# 2. Scope

Operating theatre of Gynaecology A. The protocol applies to physicians and residents in anaesthesiology and intensive care, obstetrics and gynaecology, pharmacists, and anaesthesia technicians assigned to the operating theatre.

# 3. Reference framework

- SFAR/SPILF formal expert recommendations on antibiotic prophylaxis in surgery and interventional medicine, 2024.
- ACOG Practice Bulletin No. 195: Prevention of Infection After Gynecologic Procedures, 2018.
- ACOG Practice Bulletin No. 199: Use of Prophylactic Antibiotics in Labor and Delivery, 2018.
- INEAS guideline on management of postpartum haemorrhage, December 2022.
- Journal of Obstetrics and Gynaecology Canada No. 275: Antibiotic Prophylaxis in Gynaecologic Procedures.
- World Health Organization. Global guidelines for the prevention of surgical site infection, second edition, 2018.
- Additional national prevalence, antimicrobial prophylaxis quality-standard, and EUCAST documents cited in the original protocol.

# 4. Definitions and target organisms

Surgical antibiotic prophylaxis is administration, before the start of a surgical or interventional procedure, of an antibiotic dose targeting the bacteria responsible for surgical-site infection, with the aim of achieving effective blood and tissue concentrations during the procedure, reducing perioperative contamination, and minimising postoperative infection risk.

A surgical-site infection is an infection occurring within 30 days after surgery, or within one year when implanted material is present, involving the incision, deep soft tissues, or an organ/space manipulated during the operation.

- Skin incision: Staphylococcus aureus and enteric flora.
- Uterine or vaginal incision: vaginal flora, including aerobic and anaerobic polymicrobial flora.

# 5. Responsibilities

Responsible units: Gynaecology; heads of Anaesthesiology and Intensive Care, Gynaecology A, and Pharmacy.

# 6. General principles

- Use prophylaxis for selected clean and clean-contaminated procedures (Altemeier classes 1 and 2).
- Select an agent with a narrow spectrum directed at the predefined bacterial target and avoid agents with local resistance >20% when an approved alternative is available.
- Consider the microbiological ecology of the anatomical site and the continuity created between anatomical compartments.
- Prefer a cost-conscious regimen.
- Decide and document the indication during the pre-anaesthetic assessment; adapt to the planned procedure, infection risk, previous antibiotic exposure, and known carriage of resistant organisms.
- Keep the protocol available and displayed in the operating theatre.
- Protocol deviations must remain exceptional and be justified.
- Adapt the regimen for multidrug-resistant-organism carriage and early non-infectious reoperation.
- When clinically relevant, screen and treat bacterial vaginosis before hysteroscopy.
- The prescriber may be the anaesthesiologist-intensivist or the surgeon. Administration is performed by the anaesthesia physician or anaesthesia technician.

# 7. Standard initial doses, timing, redosing, and duration

| **Procedure / indication** | **Recommended agent** | **Initial dose / route** | **Redosing and duration** |
| --- | --- | --- | --- |
| Standard prophylactic administration | Administer approximately 30 minutes before incision, including planned caesarean delivery, and as close as practicable to incision or procedure start. |  |  |
| Cefazolin | Cefazolin | 2 g IV over approximately 30 min | Repeat 1 g every 4 h when indicated |
| Amoxicillin-clavulanate | Amoxicillin-clavulanate | 2 g/200 mg IV | For prolonged surgery, alternate 1 g amoxicillin with 1 g amoxicillin-clavulanate every 2 h to limit clavulanate exposure |
| Ceftriaxone | Ceftriaxone | 2 g IV | Single dose; no routine intraoperative redosing |
| Cefotaxime | Cefotaxime | 2 g IV | Repeat 1 g every 2 h when indicated |
| Gentamicin | Gentamicin | 5 mg/kg IV; maximum 500 mg in the general regimen | Single dose |
| Clindamycin | Clindamycin | 900 mg IV | Repeat 450 mg every 4 h when indicated |
| Metronidazole | Metronidazole | 1 g IV | Single dose |
| Doxycycline | Doxycycline | 200 mg orally | A second 200-mg oral dose may be given at the end of the procedure when specified |

Prophylaxis beyond 48 hours is prohibited. The presence of a surgical drain does not justify extending prophylaxis.

# 8. Special situations

- Obesity: routine cephalosporin dose escalation is not recommended solely to reduce SSI, except in specific situations such as very high body mass index or body weight >120 kg, when dose doubling may be considered.
- For beta-lactam-allergic patients with obesity, the original protocol proposes clindamycin 900 mg for BMI 30-45 kg/m², 1,200 mg for BMI 45-60 kg/m², and 1,600 mg for BMI >60 kg/m²; gentamicin 6-7 mg/kg using adjusted body weight.
- Markedly increased distribution volume: consider redosing when blood loss exceeds 1,500 mL.
- Immunosuppression, radiotherapy, chemotherapy, corticosteroid therapy, poorly controlled diabetes, advanced age, obesity, or low body weight do not justify disregarding the general prophylaxis rules.

# 9. Obstetric surgery recommendations

| **Procedure / indication** | **Recommended agent** | **Initial dose / route** | **Redosing and duration** |
| --- | --- | --- | --- |
| Caesarean delivery, planned or emergency; uterine-body suture for obstetric rupture | Cefazolin or ceftriaxone or cefotaxime Beta-lactam allergy: clindamycin | Cefazolin 2 g IV slowly Ceftriaxone 2 g IV Cefotaxime 2 g IV Clindamycin 900 mg IV over 30 min | Cefazolin: repeat 1 g every 4 h if duration >4 h Ceftriaxone: single dose Cefotaxime: repeat 1 g every 2 h if duration >2 h Clindamycin: repeat 450 mg every 4 h if duration >4 h |
| Cervical cerclage during pregnancy; removal of cervical cerclage | No prophylaxis | — | — |
| Manual placental removal / artificial delivery; uterine revision after vaginal delivery; manual extraction of a complete placenta | Cefazolin or ceftriaxone or cefotaxime or amoxicillin-clavulanate Beta-lactam allergy: clindamycin + gentamicin | Cefazolin 2 g IV slowly Ceftriaxone 2 g IV Cefotaxime 2 g IV Amoxicillin-clavulanate 2 g/200 mg IV Clindamycin 900 mg IV + gentamicin 5 mg/kg IV | Cefazolin: repeat 1 g q4h if >4 h Ceftriaxone: single dose Cefotaxime: repeat 1 g q2h if >2 h Amoxicillin-clavulanate: alternate amoxicillin / amoxicillin-clavulanate q2h if prolonged Clindamycin: repeat 450 mg q4h if >4 h; gentamicin single dose |
| Haemostatic hysterectomy for obstetric complications by laparotomy | Same options as for manual placental removal / uterine revision | Same initial doses | Same redosing rules |
| Vascular ligation for postpartum haemorrhage by laparotomy, including internal iliac artery or uterine pedicle ligation | Cefazolin or ceftriaxone or cefotaxime Beta-lactam allergy: clindamycin | Cefazolin 2 g IV slowly Ceftriaxone 2 g IV Cefotaxime 2 g IV Clindamycin 900 mg IV over 30 min | Cefazolin q4h if >4 h; ceftriaxone single dose; cefotaxime q2h if >2 h; clindamycin q4h if >4 h |

# 10. Gynaecological surgery: hysteroscopic, laparoscopic, adnexal, and endometriosis procedures

| **Procedure / indication** | **Recommended agent** | **Initial dose / route** | **Redosing and duration** |
| --- | --- | --- | --- |
| Hysteroscopy: endometrial destruction/ablation, therapeutic curettage, polypectomy, endometrial biopsy, myomectomy | No routine prophylaxis. In selected patients with previous upper genital-tract infection, the original protocol provides a risk-adapted regimen; when a positive vaginal sample is also present, doxycycline may be added. | Risk-adapted regimen in the original document: cefazolin 2 g; if positive vaginal sample, cefazolin 2 g + doxycycline 200 mg orally; beta-lactam allergy: clindamycin 900 mg + doxycycline 200 mg. | Single perioperative regimen unless otherwise stated |
| Termination of pregnancy / uterine evacuation after abortion or non-viable pregnancy | Doxycycline | 200 mg orally 1 h before the procedure and 200 mg orally after the procedure | Two-dose regimen |
| Diagnostic laparoscopy; tubal ligation; tuboplasty; ovarian drilling, cystectomy, or transposition; cyst puncture; removal of intrauterine device | No routine prophylaxis; apply the same upper-genital-tract-infection risk exceptions as for hysteroscopy | — | — |
| Adnexectomy; treatment of pelvic or lumbar hernia/omental procedure | Cefazolin or ceftriaxone or cefotaxime Beta-lactam allergy: clindamycin | Cefazolin 2 g IV slowly Ceftriaxone 2 g IV Cefotaxime 2 g IV Clindamycin 900 mg IV over 30 min | Cefazolin q4h if >4 h; ceftriaxone single dose; cefotaxime q2h if >2 h; clindamycin q4h if >4 h |
| Resection of endometriotic lesions involving the rectum or rectovaginal septum | Cefazolin, ceftriaxone, or cefotaxime + metronidazole or amoxicillin-clavulanate Beta-lactam allergy: clindamycin + gentamicin | Cephalosporin 2 g IV + metronidazole 1 g IV Amoxicillin-clavulanate 2 g/200 mg IV Clindamycin 900 mg IV + gentamicin 5 mg/kg IV | Agent-specific redosing as above; metronidazole and gentamicin single dose |

# 11. Gynaecological surgery: laparotomy and uterine surgery

| **Procedure / indication** | **Recommended agent** | **Initial dose / route** | **Redosing and duration** |
| --- | --- | --- | --- |
| Adnexal surgery by laparotomy: tubal surgery, adnexectomy, ovariectomy, treatment of pelvic/lumbar hernia or omental procedure | Cefazolin or ceftriaxone or cefotaxime Beta-lactam allergy: clindamycin | Cefazolin 2 g IV; ceftriaxone 2 g IV; cefotaxime 1-2 g IV according to the original table; clindamycin 900 mg IV | Cefazolin q4h if >4 h; ceftriaxone single dose; cefotaxime q2h if >2 h; clindamycin q4h if >4 h |
| Excision of endometriotic lesions of the rectovaginal septum by laparotomy | Ceftriaxone or cefotaxime + metronidazole or amoxicillin-clavulanate Beta-lactam allergy: clindamycin + gentamicin | Ceftriaxone/cefotaxime 2 g IV + metronidazole 1 g IV Amoxicillin-clavulanate 2 g/200 mg IV Clindamycin 900 mg IV + gentamicin 5 mg/kg IV | Cephalosporin/amoxicillin-clavulanate/clindamycin redosing according to half-life; metronidazole and gentamicin single dose |
| Total hysterectomy ± adnexectomy; radical colpohysterectomy; radical trachelectomy, by laparotomy or laparoscopy | Amoxicillin-clavulanate or ceftriaxone/cefotaxime + metronidazole Beta-lactam allergy: clindamycin + gentamicin | Amoxicillin-clavulanate 2 g/200 mg IV Ceftriaxone or cefotaxime 2 g IV + metronidazole 1 g IV Clindamycin 900 mg IV + gentamicin 5 mg/kg IV | Agent-specific redosing; metronidazole and gentamicin single dose |
| Subtotal hysterectomy ± adnexectomy without vaginal phase; myomectomy; isthmic cerclage outside pregnancy; removal of intrauterine device | Cefazolin or ceftriaxone or cefotaxime Beta-lactam allergy: clindamycin | Cefazolin 2 g IV; ceftriaxone 2 g IV; cefotaxime 2 g IV; clindamycin 900 mg IV | Agent-specific redosing as above |

# 12. Vaginal surgery, prolapse surgery, and breast surgery

| **Procedure / indication** | **Recommended agent** | **Initial dose / route** | **Redosing and duration** |
| --- | --- | --- | --- |
| Vaginal uterine procedures: conisation; curettage or endometrial destruction; vaginal isthmic cerclage outside pregnancy; cervical lesion destruction; adhesiolysis; myomectomy | No routine prophylaxis. Apply risk-adapted upper-genital-tract-infection exceptions described for hysteroscopy. | — | — |
| Vaginal hysterectomy ± adnexectomy; subtotal or total colpectomy | Amoxicillin-clavulanate or ceftriaxone/cefotaxime + metronidazole Beta-lactam allergy: clindamycin + gentamicin | Amoxicillin-clavulanate 2 g/200 mg IV Ceftriaxone 2 g or cefotaxime 1-2 g IV + metronidazole 1 g IV Clindamycin 900 mg IV + gentamicin 5 mg/kg IV | Agent-specific redosing; metronidazole and gentamicin single dose |
| Prolapse surgery, any approach: hysteropexy, promontofixation, colpoperineorrhaphy | Cefazolin or ceftriaxone or cefotaxime Beta-lactam allergy: clindamycin | Cefazolin 2 g IV; ceftriaxone 2 g IV; cefotaxime 2 g IV; clindamycin 900 mg IV | Agent-specific redosing as above |
| Partial or total mastectomy, with or without axillary dissection, with or without immediate reconstruction; breast lumpectomy with axillary dissection | Cefazolin or ceftriaxone or cefotaxime Beta-lactam allergy: clindamycin | Cefazolin 2 g IV; ceftriaxone 2 g IV; cefotaxime 2 g IV; clindamycin 900 mg IV | Agent-specific redosing as above |
| Breast lumpectomy without axillary dissection; lumpectomy with sentinel-node procedure | No prophylaxis | — | — |

# 13. Monitoring and review

The effectiveness of the prophylaxis protocol should be reassessed regularly by monitoring surgical-site infection rates and causative microorganisms, and by periodic evaluation of professional practice.

# 14. Comparative overview versus the baseline written protocol

| **Domain** | **Baseline protocol** | **Revised protocol** |
| --- | --- | --- |
| Scope | Hospital-wide protocol with a short obstetric-gynaecological section | Dedicated 17-page obstetric-gynaecological protocol |
| Reference base | Primarily SFAR 2018 | SFAR/SPILF 2024 plus obstetric-gynaecological and SSI guidance |
| Governance | No dedicated version-control or multidisciplinary signature framework documented in the baseline extract | Version 01; named drafters; formal validation by Anaesthesiology and Gynaecology service heads |
| Responsibilities | Not clearly allocated in the baseline departmental use | Prescriber and administrator roles explicitly described |
| Clinical content | Limited procedure categories and fewer alternatives | Expanded procedure-specific tables, redosing rules, risk exceptions, obesity guidance, and surveillance provisions |
| Implementation | Available as a general hospital reference | Designed for point-of-care dissemination, training, role clarification, and practice monitoring |
